# Study on the skid resistance performance of wet and slippery asphalt pavement on curved roads in aeolian sand areas

**DOI:** 10.1371/journal.pone.0332033

**Published:** 2025-09-15

**Authors:** Qiang Pan, Ping Li, Xiaoyan Liu, Jingsheng Pan

**Affiliations:** Department of Civil Engineering, Chengdu Technological University, Yibin, China; Shandong University of Technology, CHINA

## Abstract

In order to study the change law of the anti sliding performance of desert highway in the wet and slippery state of the curve, two different curves and straight roads of the same highway were selected for the field test through the method of field test. The British pencil number (BPN) and mean texture depth (MTD) were selected as the evaluation indexes, and the change law of BPN and MTD at different curves and straight roads with the amount of sand and water was measured. In order to clarify the sensitivity of the anti sliding performance under different humidity, the water volume (humidity) that has the greatest impact on BPN and MTD was obtained by using grey correlation analysis, and then the anti sliding performance at the curves and straight roads was evaluated.The performance is compared and analyzed. The results show that the anti sliding performance of the curve and the straight road decreased by 33.3% and 32.0%, respectively, and the change law of the two is basically the same. The water aggravates the decline of the anti sliding performance of the road surface, and the anti sliding performance of the curve at low speed is about 3.2% less than that of the straight road.

## 1. Introduction

Asphalt pavement skid resistance is a critical indicator for ensuring road traffic safety and enhancing pavement performance. In terms of traffic safety, skid resistance directly affects vehicle braking efficiency and driving stability. Insufficient friction coefficients can extend braking distances by over 30% under wet conditions, significantly increasing risks of rear-end collisions, skidding, and other accidents. Statistics indicate that approximately 40% of rainy-day traffic accidents are directly linked to inadequate pavement skid resistance [1–3]. From a pavement performance perspective, appropriate texture depth not only facilitates effective drainage of surface water films but also enhances mechanical interlock between tires and the pavement, mitigating rutting deformation and aggregate wear caused by heavy-duty vehicles. In critical areas such as sharp curves and long slopes in mountainous regions, sustained skid resistance stability can reduce structural pavement damage risks by more than 50%.

At present, the research on the resistance performance of asphalt pavement mainly focuses on the friction coefficient and surface texture. Good road surface texture provides sufficient adhesion for tires to ensure safe driving of vehicles [4–8]. Marcelo [9] and others established the three-dimensional structure of the pavement through the close range photogrammetry technology, and then obtained the macro parameters. Mahboob [10] confirmed the relationship between macro texture change and anti sliding value based on actual working conditions through field tests. Dai [11] collected three-dimensional texture data from the new asphalt pavement, used discrete Fourier transform to separate the macro and micro texture, and effectively realized the evaluation of pavement skid resistance through the machine evaluation model.

There are many factors that affect the skid resistance of pavement, including pavement structure, material, tire pattern, temperature, traffic load, vehicle speed and pavement pollutants. In the past, most studies focused on the lack of skid resistance caused by pavement materials and structure [12–16]. Kane [17] conducted polishing tests on samples of different pavements in the laboratory, conducted mineralogical analysis on the coarse aggregate of the samples, and proposed a new parameter of “average aggregate hardness parameter”, which is closely related to the long-term skid resistance of the pavement. Wang [18] studied two kinds of steel slag and two kinds of conventional aggregate as samples, and believed that steel slag has good wear resistance and richer micro texture, and can be used as a promising substitute for aggregate of pavement wearing course. When the road is directly exposed to the natural environment, the texture of the road surface will be more or less affected by rainfall, snow, ice and pollutants, which reduce the depth of the pavement structure and even hinder the direct contact between the wheel and the pavement, leading to a significant decline in the skid resistance [19–22]. Xiao [23] studied the influence of pollutant particles on the skid resistance of pavement, and believed that the particle size that determines the lowest friction level gradually increases with the increase of surface roughness, and the friction force first decreases sharply and then increases slowly with the increase of pollutant particle coverage. Zhu [24] studied the influence of wet and slippery road conditions on the skid resistance of runway. It can be seen that the existence of most pollutants will affect the skid resistance of pavement.

In desert regions, however, wind-driven sand accumulation on pavement surfaces introduces additional mechanical abrasion between tires and sand particles, accelerating skid resistance deterioration [25–27]. Li [28] investigated texture changes and skid resistance impacts under aeolian sand coverage. Notably, previous studies in arid desert environments primarily focused on dry sand effects, overlooking skid resistance variations under wet conditions—despite occasional rainfall in these areas. Therefore, this paper mainly through the field test, taking into account the rainfall conditions, tests the influence of aeolian sand on the surface texture and pavement friction coefficient under wet conditions, and then summarizes the anti sliding performance decay law of aeolian sand pavement under wet conditions.

## 2. Experimental conditions and methods

### 2.1. Experimental conditions

**Fig 1:**
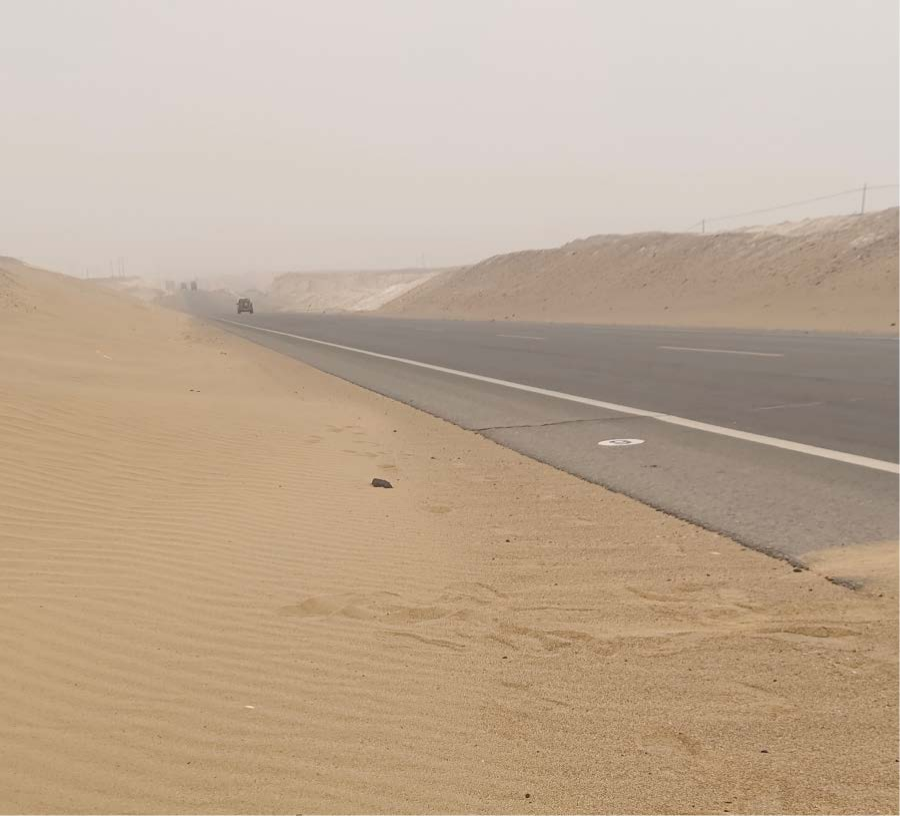
Realistic View of Desert Highway.

In this test, a class II Highway located in Xinjiang, China, is selected as the test section. The surface course is asphalt mixture pavement, and the longitudinal slope of the pavement is not more than 5%. The curve with a horizontal curve radius of about 1000m is selected as the curve. Because the desert area is relatively flat, there is no sharp curve and other special linearity, and the highway alignment is mainly long straight line and large radius horizontal curve. In addition, horizontal and straight sections are selected for comparison. Selecting a single typical radius (1000m) helps to ensure comparability between different curve segments and between curve and straight line results, and avoid introducing too much complexity due to large radius differences. The test sections are located in desert areas, with different degrees of sand accumulation on both sides of the road, and no sand prevention facilities, as shown in Fig 1. In order to ensure the stability of the field test results, the field test was carried out in windless weather.

### 2.2. Testing methods

In the test section, two corners and two straight sections are selected for comparison. The test is conducted in the morning. There are fewer vehicles in this time period, and the temperature change is relatively small, so as to reduce the interference caused by temperature difference as much as possible. In addition, all tests are conducted in windless weather, and each test section (straight line/curve) completes data collection within the same windless day to avoid wind fluctuation interference.In this study, the mass (g) is used as the measurement standard in the simulation of sand (sand) and wet (water). The porosity of aeolian sand (30% ~ 50%) is significantly affected by particle size, shape and humidity. Under the same volume, the mass fluctuation can reach ± 25%. In field operation, the mass method can achieve high repeatability through standard spreading and precision balance (± 0.01g), while the volume method has poor repeatability due to disturbance. For the simulation of wet state, the quality control humidity is still used. Because the mass method is irreplaceable, the microbalance (± 0.001g) is used to weigh the water quality, and the uniform distribution is achieved through atomization spraying. It is easy to achieve in the experiment, and has high controllability.

The pendulum friction coefficient instrument is used to measure the BPN value under different sediment deposition conditions. During the test, a fixed area of 300 × 300 mm is selected, and the different sediment deposition degrees are simulated by the sediment deposition amounts of 0g, 10g, 20g, 30g, 40g, 50g, 60g, 70g and 80g respectively. The BPN value was tested three times under each sediment volume, and the average value was taken at last. Based on this sediment volume, the MTD of different curves and straight channels were tested by sand laying method. It is worth noting that the influence of wet and slippery conditions on BPN value and MTD should also be considered in the test. Water is sprayed in the measurement area of 100 × 100 mm with spray pots, and the spray quality is 5g, 10g, 15g and 20g respectively. On the one hand, under the typical rainfall intensity and medium and low speed driving conditions, the dynamic water film thickness on the asphalt pavement is usually between 0.5 mm and 2.0 mm, so the water volume is 5g, 10g, 15g and 20g respectively according to the conversion of spray area. On the other hand, the single rainfall in the desert is about 5−15 mm. In this experiment, the rainfall is set from 5g in the area of 100*100 mm.Spraying, according to the conversion, the rainfall is 0.5 mm, which can better simulate the transition of the road from dry to wet. Moreover, this gradient design (with an interval of 5g) can clearly reveal the sensitivity and trend of anti sliding performance (such as friction coefficient) with the change of water volume, and provide sufficient data points for model establishment or performance evaluation. In the simulation of sediment deposition and humidity, the quality is used to measure, rather than volume or density. The main reason is that the quality is more intuitive, and it is easier to operate in the field test, so as to reduce the experimental error. The calibration of pendulum friction coefficient instrument and the test process of sand paving method shall be carried out in accordance with the code for field test of highway subgrade and pavement (JTG 3450−2019) [29].

## 3. Results and discussion

### 3.1. Anti slip performance at dry bends

**Fig 2:**
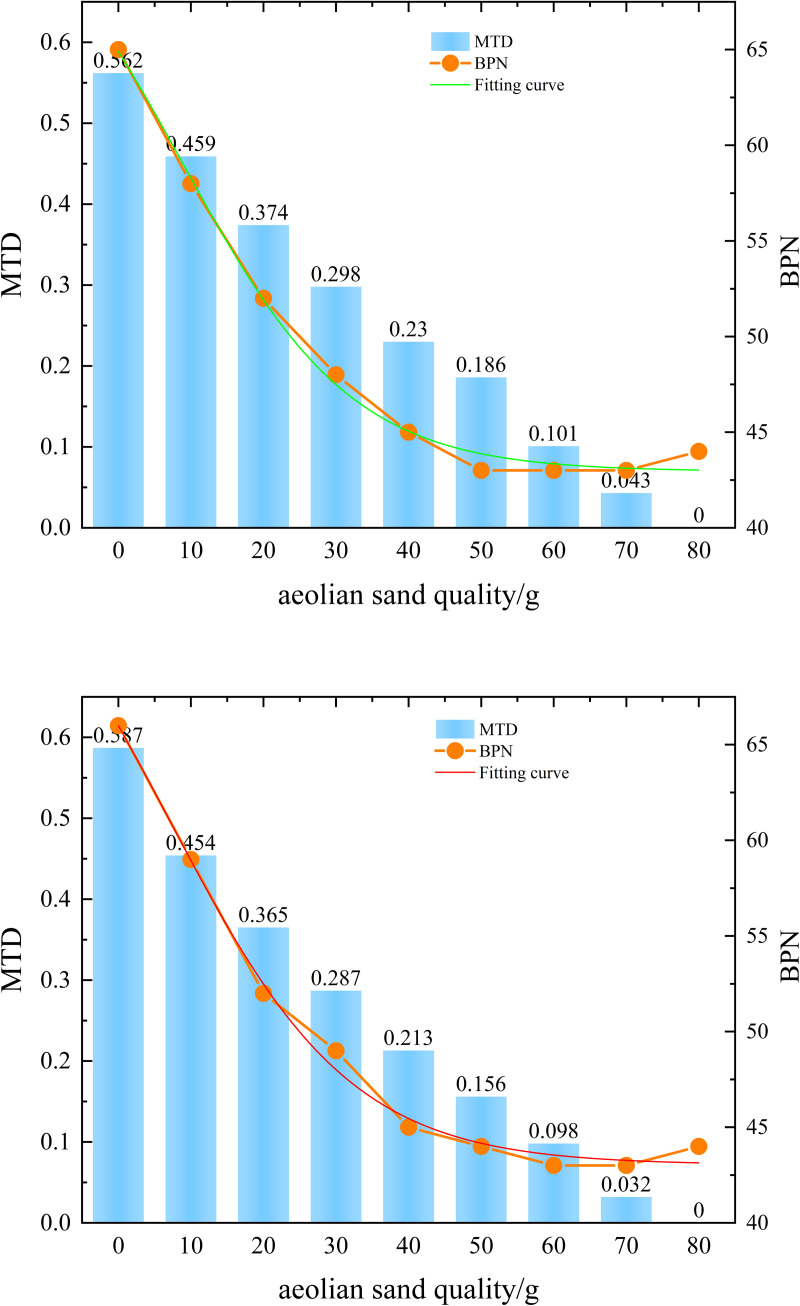
Test results at the bend.

In order to investigate the changes in anti-skid performance of wind blown sand asphalt pavement at bends, two bends were selected for field tests. Different sand accumulation amounts were used to simulate different sand accumulation situations. The BPN values and MTD under different sand accumulation amounts are shown in Fig 2. When testing, select a fixed area and simulate different sand accumulation amounts by increasing the sand weight in increments of 10g in the fixed area.

As shown in Fig 2, the changes of BPN and MTD with sediment deposition at the two bends are shown respectively, and the BPN value is fitted. On the whole, BPN and MTD both decreased sharply at first and then slowly with the increase of sediment deposition, and the two bends showed the same change rule. Among them, the BPN value showed a three-stage downward trend, which decreased sharply in the early stage, then decreased slowly, and finally tended to a stable state. MTD showed a two-stage downward trend, with a large decline in the early stage, and then decreased. In addition, the change of BPN value was fitted and analyzed by doseresp, and the correlation was 0.99. The sand and water increment (“dose”) and BPN attenuation (“response”) showed nonlinear saturated attenuation characteristics, which was in line with the core assumption of doseresp model.

From Fig 2, it can be seen that the MTD decreases rapidly when the sand accumulation increases to 20g in the early stage, decreasing by 33.5% and 37.8% respectively. Then the decrease slightly decreases, and the six gradient growth of sand accumulation reduces the MTD by 88.5% and 90.2%. When the sand accumulation increases to 80g, the MTD is 0. During testing, we noticed that the texture of this accumulated road surface is almost covered. We believe that the reason for this phenomenon is that as the amount of accumulated sand increases, more and more voids are filled. For BPN, the first stage is when the sediment accumulation increases to 20g, with a decrease of 20.1% and 21.2% respectively. The second stage is when the sediment accumulation increases from 20g to 50g, with a decrease of 17.3% and 15.4% respectively. In the final stage, BPN tends to stabilize and even shows a slight rebound. Analysis suggests that the rapid decrease in BPN value in the early stage is due to the presence of a small amount of sand particles, which formed a micro bearing system between the rubber slider, sand particles, and road surface, resulting in a transition from sliding friction to rolling friction. The slight increase in BPN value in the later stage is due to the limitations of the pendulum friction coefficient meter. The increase in sand particles has a certain buffering effect on the pendulum, so it will rise slightly at this stage.

### 3.2. Anti slip performance at wet and slippery bends

**Fig 3:**
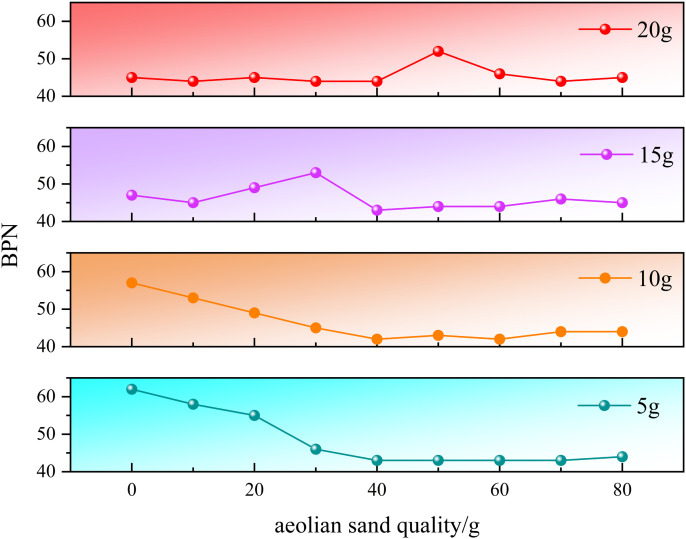
BPN values at wet and slippery bends.

**Fig 4:**
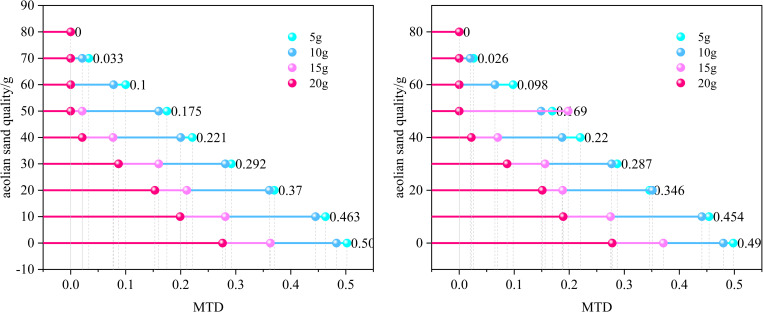
MTD values at wet and slippery bends.

Due to the perennial drought and lack of rainfall in desert areas, there has been little attention paid to the wet sliding condition in previous studies on the anti-skid properties of asphalt pavements with graded sand, which is the main focus of this article. BPN and MTD were tested at different humidity levels on the previously selected test section, and the test results are shown in Figs 3 and 4.

As shown in Fig 3, the changes in BPN with sediment accumulation and humidity at two bends are shown. The road surface humidity is 5g, 10g, 15g, and 20g, respectively. Overall, the trend of change at the two bends is basically the same, and as the sediment accumulation increases, the BPN value decreases overall. The presence of accumulated water on the road surface exacerbates this trend. There are significant differences in BPN values under different water accumulation amounts. When the water accumulation amount is 5g, the decrease in BPN value is mainly concentrated in the early stage. When the sand accumulation amount increases from 0g to 20g, the BPN value decreases slowly, decreasing by 12.9% and 11.3% respectively. When the sand accumulation amount increases from 20g to 40g, the rate of BPN value decrease increases, decreasing by 20.4% and 20.0% respectively, and reaching the lowest point before stabilizing. Moreover, the same variation pattern was observed when the accumulated water volume was 10g. We believe that at this point, moisture occupies some of the gaps, causing more sand to cover the road surface texture, and the upper layer of sand particles is more moist, playing a lubricating role. However, when the accumulated water amount increased to 15g, different patterns of change were observed. Specifically, the BPN value had already reached a relatively low level and did not show significant changes with the increase of sediment accumulation. Only when the sediment accumulation was 30g, the curve showed obvious fluctuations. Analysis suggests that at this point, the moisture is just enough to provide cohesive force for the accumulated sand, increasing the overall integrity of the road surface windblown sand. The rubber slider of the pendulum friction coefficient meter experiences resistance when it falls, resulting in an increase in BPN value. However, as the amount of accumulated sand increases, the moisture is insufficient to provide cohesive force for the accumulated sand, causing the BPN value to decrease again to its previous state. This rule still applies when the accumulated water volume increases to 20g, but the fluctuation of BPN value is relatively delayed at this time, because in order to make wind blown sand have a certain degree of cohesion under this accumulated water volume, the accumulated sand volume also needs to be increased accordingly.

As shown in Fig 4, the MTD at two bends varies with sediment accumulation and humidity, and the road surface humidity remains at 5g, 10g, 15g, and 20g, respectively. The trend of change at the two bends remains the same. When the accumulated water volume is 5g, the impact on MTD is relatively small. At this time, the decrease in MTD is 7.7% and 8.8% respectively. Moreover, under the same sediment accumulation, the decrease in MTD is also very small as the accumulated water volume increases from 5g to 10g. We believe that the accumulated water at this time is not enough to completely fill the gaps on the road surface, and as the amount of accumulated sand increases, the role of water gradually decreases. When the amount of accumulated water increases to 15g, the decrease in MTD significantly increases. At this time, most of the road surface gaps are filled with accumulated sand and water, and the more water increases, the more gaps are occupied. Therefore, the decrease in MTD becomes more pronounced. The amount of sediment accumulation at which the MTD value reaches a stable state varies depending on the amount of accumulated water. For example, when the accumulated water is 5g, the MTD tends to stabilize at a sediment accumulation of 70g, when the accumulated water is 10g, the MTD tends to stabilize at a sediment accumulation of 60g, when the accumulated water is 15g, the MTD tends to stabilize at a sediment accumulation of 50g, and when the accumulated water is 20g, the MTD tends to stabilize at a sediment accumulation of 40g.The analysis shows that the dense pores formed between sand particles can hold more water with the increase of sand volume, which leads to the water being limited in the particle gap and unable to migrate to the sand road interface.

According to the above test results, the influence degree of different ponding volume on BPN and MTD varies. In order to further study the sensitivity of BPN and MTD under different ponding volume, the above results were analyzed by grey correlation analysis, which can overcome the limitation of small samples, be compatible with multi-source heterogeneous data, and accurately quantify the independent and interactive effects of complex factors such as sand and water. The results of correlation order can provide priority basis for anti sliding performance maintenance.

**Fig 5:**
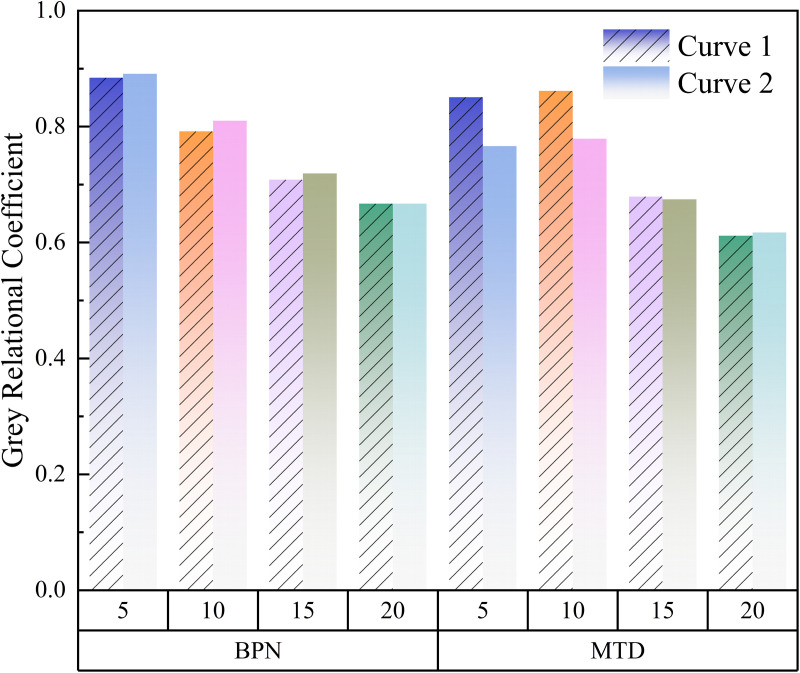
Grey correlation coefficient for different water accumulation amounts.

In the grey correlation analysis of MTD, the MTD measured at bend 1 and bend 2 in the conventional state is taken as the reference sequence, and the MTD at different ponding volumes of bend 1 and bend 2 is taken as the comparison sequence. In the grey relational analysis of BPN, the BPN measured at bend 1 and bend 2 in the conventional state is taken as the reference sequence, and the BPN measured at different ponding volumes of bend 1 and bend 2 is taken as the comparison sequence. Moreover, in order to eliminate dimensional differences and fairly compare the sensitivity of BPN and MTD to ponding depth, the original data were normalized by the averaging method, and then the grey correlation coefficient was calculated. The results are shown in Fig 5.

Fig 5 shows the grey correlation coefficient of BPN and MTD under different humidity. It can be seen from the figure that when the amount of accumulated water is 5g, the grey correlation coefficient of BPN is the largest, which means that BPN is the most sensitive under this humidity. The analysis shows that the existence of water, although few, still changes the state of aeolian sand, from dry to wet, and the capillary liquid bridge formed by 5g water breaks instantaneously at the initial stage of shear, leading to the collapse of the sand bite structure and the sharp change of BPN, so it has the greatest impact on BPN. When the ponding volume is 10g, the grey correlation coefficient of MTD is the largest, indicating that the impact on MTD is the largest when the ponding volume is 10g. At this time, water occupies part of the gap of the pavement, and then water gradually infiltrates into the aeolian sand on the road surface through capillary action, so the impact degree is reduced. We note that the grey correlation coefficient of MTD is similar when the water volume is 5g and 10g, in which the grey correlation coefficient is 0.8508 and 0.7663 when the water volume is 5g, and 0.8616 and 0.7792 when the water volume is 10g. When the amount of accumulated water increases to 15g and 20g, the grey relational number is relatively small. At this time, although the amount of water increases, the increased water is mainly immersed into the aeolian sand, so the impact is small.

**Fig 6:**
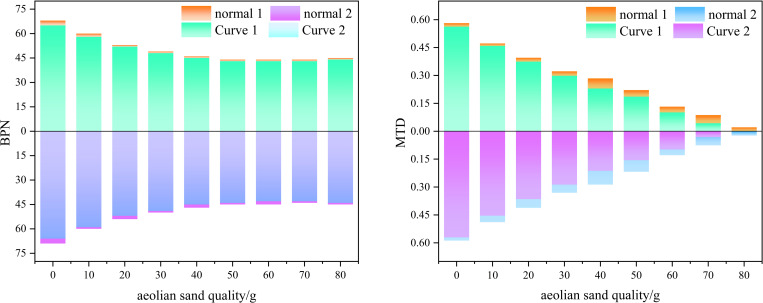
Comparison of Parameters between Curves and Straight Roads.

Overall, the grey correlation coefficient of BPN is greater than MTD. Therefore, the maximum grey correlation coefficient in BPN corresponding to a water accumulation of 5g was selected to compare and analyze the BPN and MTD at bends and straight roads under this humidity. Moreover, the grey correlation coefficient of MTD is not significantly different when the water accumulation is 5g and 10g, with a difference of only about 0.1. The results considered from both aspects are similar. Therefore, a comparative analysis was conducted on the BPN and MTD at the bend and straight road when the water accumulation is 5g. The results are shown in Fig 6.

As shown in Fig 6, when the water accumulation is 5g, the BPN and MTD at the bend and straight sections are compared. Overall, the BPN value at the straight section is slightly higher than that at the bend, and the difference is very small. Although some friction forces need to overcome centrifugal forces when driving on bends, leading to a mistaken belief that the anti-skid performance at bends is reduced, in reality, the vehicle’s speed at bends is relatively low, resulting in less centrifugal force and less wear on the road surface. Therefore, the BPN at bends and straightaways is almost the same. In addition, the limitations of the testing method are another reason for such results. BPN testing is based on the sliding friction of the pendulum at static or low speeds, making it difficult to fully simulate the dynamic friction requirements of the vehicle during high-speed cornering. The actual dynamic friction force on bends may be weakened by the standardized conditions of the testing method compared to straight roads, resulting in insignificant numerical differences. The difference in MTD between the curved and straight sections is relatively small in the early stage. As the sediment accumulation increases, the difference in MTD gradually increases. In the last two cases, the MTD is almost zero, and the difference is also almost zero. Due to the shear force generated when vehicles turn at bends, more wind blown sand particles enter the gaps on the road surface. Therefore, compared to straight roads, the MTD at bends is slightly smaller. This phenomenon is more pronounced when the sand accumulation is 40g and 50g. This is because the wind blown sand amount is relatively small in the early stage, and the gaps on the road surface are relatively rich, which can naturally accommodate some sand particles. Therefore, the difference in MTD is small compared to straight roads. Later, as the sand accumulation increases, the gaps on the road surface are gradually filled. At this time, under the action of shear force, some sand particles are passively squeezed into the gaps on the road surface, resulting in a relative increase in the difference in MTD.

## 4. Conclusions

Given the perennial arid climate of desert regions, where wet conditions are rarely considered in road skid resistance studies, this research conducted field experiments to evaluate curve-section skid resistance under wet-slippery conditions using British Pendulum Number (BPN) and Mean Texture Depth (MTD) as evaluation metrics. The findings are summarized as follows:

(1) The declining trends of BPN and MTD at curve sections aligned with previous findings for straight sections [25]. Notably, BPN values exhibited minor rebounds in later stages, a phenomenon attributed to the limitations of the pendulum friction coefficient tester. This observation aligns with real-world scenarios—for instance, sand spreading on icy roads enhances vehicle safety.
(2) Aeolian sand exhibited high sensitivity to water, which exerted dual effects: Firstly, water lubricated the sand particles, accelerating the decline in skid resistance; however, on the other hand, water enhanced interparticle cohesion among the sand grains, resulting in a temporary partial recovery of skid resistance during specific phases.
(3) According to the results of grey correlation analysis, the water accumulation of 5g has the greatest impact on the anti sliding performance at the bend. Therefore, during road maintenance and daily cleaning, attention should be paid to the road surface humidity at the bend, and drainage and sand cleaning measures should be taken to ensure driving safety.

In terms of road maintenance policy, it is recommended to adopt monthly BPN detection and MTD detection for high-risk sections, and increase the critical values of the two indicators accordingly. When the critical value is reached, the sand in the road area shall be cleared in time to ensure the traffic safety of the road. In terms of pavement design, the broken graded asphalt mixture (2.36 mm sieve passing rate ≤ 28%) is used to increase the structural depth to accommodate sand particles, and good drainage effect can be achieved; The curve section shall be widened accordingly, and the shoulder shall be provided with a sand guide groove.

## Supporting information

S1 Data Supporting Information. (XLSX)
